# Membrane‐type I metalloproteinase (MT1‐MMP): A key modifier of extracellular matrix microenvironment

**DOI:** 10.1111/febs.70532

**Published:** 2026-03-31

**Authors:** Yoshifumi Itoh, Masaki Inada

**Affiliations:** ^1^ Kennedy Institute of Rheumatology University of Oxford UK; ^2^ Institute of Global Innovation Research Tokyo University of Agriculture and Technology Japan; ^3^ Department of Biotechnology and Life Science Tokyo University of Agriculture and Technology Japan

**Keywords:** collagen, ECM, microenvironment signal, MT1‐MMP

## Abstract

Extracellular matrix (ECM) is a major component of the cellular microenvironment. It holds cells, directly sends signals to the cells, pools growth factors and cytokines, and guides cell migration and differentiation. A type I transmembrane proteinase, membrane‐type I matrix metalloproteinase (MT1‐MMP/MMP14), degrades ECM at the cell surface and cleaves cell surface receptors, thereby modifying cellular behavior. It is a major promoter of cell invasion and tissue destruction in diseases, such as cancer and arthritis, and is a crucial enzyme that maintains fibrillar collagen homeostasis in stromal tissue. This review discusses the current understanding of the function and regulation of this important cell surface proteinase in health and disease.

## Graphical abstract

MT1‐MMP: A cellular microenvironment modifier. MT1‐MMP is a major cellular microenvironment modifier, maintaining collagen homeostasis by degrading excess collagen (A), creating a migration path in motile cells such as cancer cells (B), modulating local microenvironmental signaling by cleaving transmembrane receptors (C), and destroying tissue architecture in diseases such as rheumatoid arthritis (D).

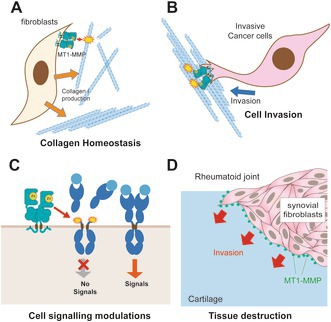

**Abbreviations**
A2M: alpha‐2‐macroglobulin
ADAM: a disintegrin and metalloprotease
ADAMTS: a disintegrin and metalloproteinase with thrombospondin motifs
BM: basement membrane
CT: cytoplasmic tail
DD: Dupuytren's disease
DDR2: discoidin domain receptor 2
ECM: extracellular matrix
EphA2: Eph receptor A2
ER: endoplasmic reticulum
ERM: Ezrin
FGFR2: fibroblasts growth factor receptor 2
Hpx: hemopexin
KIF: kinesin superfamily proteins
L1: linker 1
L2: linker 2
LRP‐1: low‐density lipoprotein receptor‐related protein 1
MMPs: Matrix metalloproteinases
MT1‐MMP: membrane‐type I matrix metalloproteinase
PCs: proprotein convertases
PTH: parathyroid hormone 1
RA: rheumatoid arthritis
Radixin: and Moesin
RTK: receptor tyrosine kinases
SNP: single nucleotide polymorphism
TIMP: tissue inhibitor of metalloproteinases
TM: transmembrane
VEGF: vascular endothelial growth factor

## Introduction

The extracellular matrix (ECM) creates a microenvironment for tissue cells. It physically holds cells within the tissue architecture, provides cells with a survival signal, serves as a reservoir for growth factors and cytokines, and guides cell migration and differentiation. Thus, ECM significantly influences cellular function and cell fate within the tissue. On the other hand, ECM is produced and organized by cells, which is crucial for maintaining proper tissue and organ function. Thus, ECM metabolism is a crucial program to maintain a steady state under physiological conditions. On the other hand, the ECM microenvironment must be modified during active tissue remodeling, such as during development and wound healing under physiological conditions, or during tissue destruction and cancer cell invasion under pathological conditions [1]. In many cases, ECM microenvironment modifications involve degradation of ECM in a spatially limited manner rather than a broader area. For instance, during tissue cell migration, ECM degradation occurs only at the leading edge of migrating cells, where it acts as a physical barrier. To achieve this, cells utilize the type I transmembrane ECM‐degrading proteinase, membrane‐type I matrix metalloproteinase (MT1‐MMP/MMP14) [2]. MT1‐MMP was originally discovered as an enzyme that promotes cancer invasion by degrading the ECM on the cell surface [3]. Since its discovery, its role in cancer invasion has been expanded to other diseases as well as its functions in physiological states. This review discusses the current understanding of MT1‐MMP‐mediated modification of the ECM microenvironment and signaling in both health and disease.

## The domain structure of MT1‐MMP

**Fig. 1:**
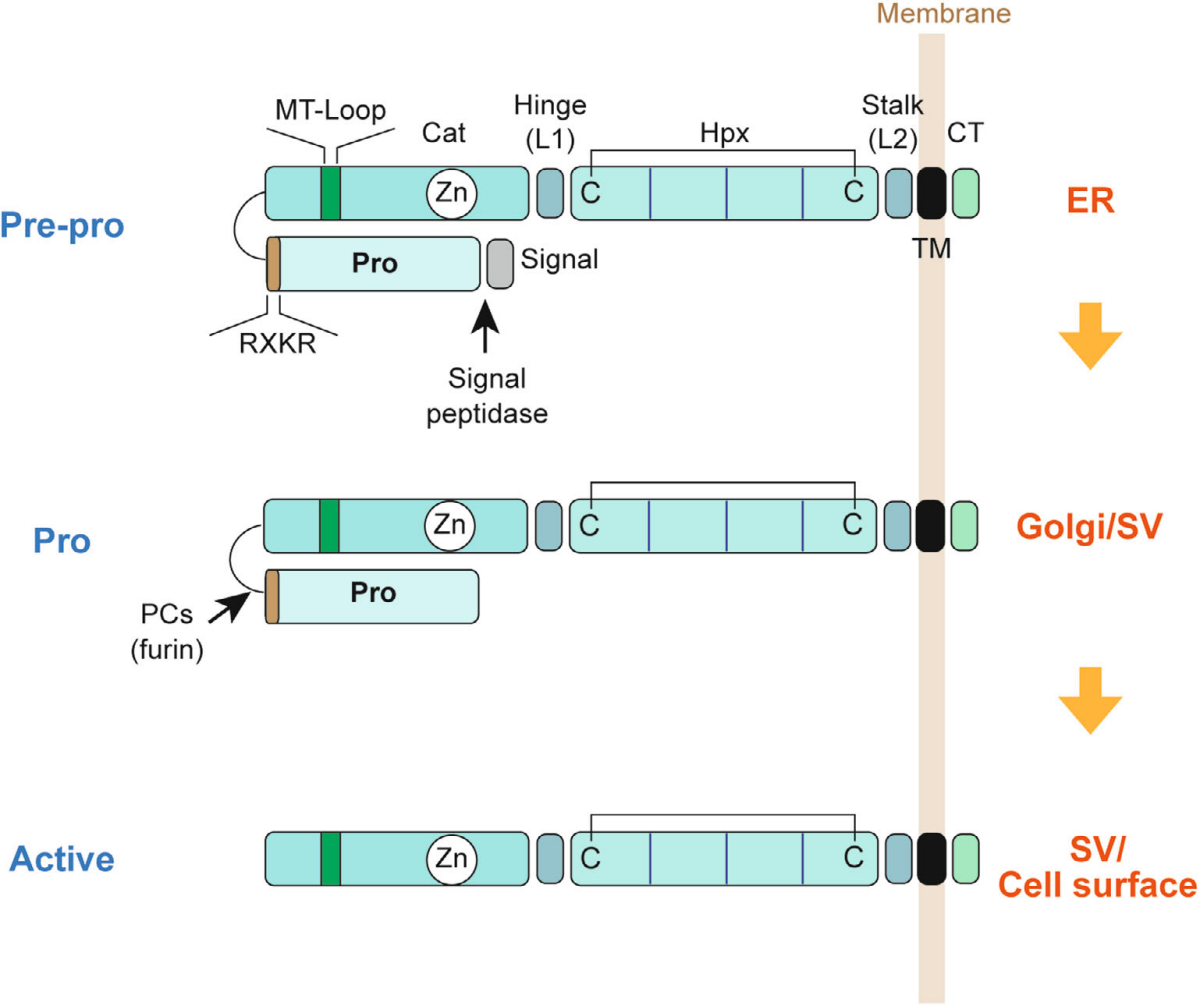
Domain structure of MT1‐MMP and its intracellular processing. MT1‐MMP is synthesized as a pre‐pro‐enzyme with a signal peptide at the N terminus of the propeptide. At the ER, the signal peptide is cleaved by a signal peptidase, converted to a pro‐enzyme. At the Golgi or during secretion within the SV, proMT1‐MMP is converted to the active form as the PCs recognize the basic motif of RRKR at the C terminus of the propeptide. It has been reported that furin is the responsible PC for this conversion. The active form of MT1‐MMP is then secreted to cell surface. Cat, catalytic domain; CT, cytoplasmic tail; ER, endoplasmic reticulum; Golgi, Golgi apparatus; Hpx, hemopexin domain; L1, linker 1; L2, linker 2; MT‐Loop, MT‐Loop region present only in the MT1‐, MT2‐, MT3‐, and MT5‐MMPs; Pro, propeptide; RXKR, Arg‐Xxx‐Lys‐Arg^112^, a motif recognized by proprotein convertases (PCs); Signal, signal peptide; SV, secretory vesicles; TM, transmembrane domain; Zn, catalytic zinc. C‐C indicates a disulfide bond bridging between the N‐ and C‐termini of the Hpx domain.

MT1‐MMP belongs to the matrix metalloproteinase (MMP) family, that are thought to be responsible for ECM degradation [4, 5, 6]. The domain structure of MT1‐MMP includes a signal peptide, a prodomain, a catalytic domain, a hinge (Linker1/L1), a hemopexin (Hpx) domain, a stalk region (L2), a transmembrane (TM) domain, and a cytoplasmic tail (CT) (Fig. 1). It is synthesized as a pre‐pro‐enzyme in the rough ER (endoplasmic reticulum), but during maturation, the signal peptide is removed by signal peptidase in the ER. Prodomain covers the catalytic site in the catalytic domain, maintaining its latency, and it must be proteolytically removed to become a functional enzyme. While many soluble MMPs are secreted from cells as proMMPs and activated extracellularly by other proteinases, some MMPs, including MMP11, MMP23, MT1‐, MT2‐, MT3‐, MT4‐, MT5‐, MT6‐MMPs are activated during secretion since the prodomain contains a basic amino acid motif of RXKR at the C terminus, which is recognized and cleaved by proprotein convertases (PCs) such as furin in the Golgi apparatus [6, 7]. It has been shown that MT1‐MMP is activated by furin [8, 9]. Thus, MT1‐MMP is secreted as a fully processed, active form before it appears on the cell surface (Fig. 1).

## Substrates of MT1‐MMP

### ECM components

MT1‐MMP cleaves various ECM components, including fibrillar collagens I, II, and III, fibronectin, vitronectin, laminins‐1, ‐2, ‐4, and ‐5, fibrin/fibrinogen, perlecan, and aggrecan [6, 10, 11, 12, 13]. MT1‐MMP is one of the five fibrillar collagen‐degrading MMPs, collagenase, and the only membrane‐bound collagenase [14]. As collagen degradation is a crucial function of MT1‐MMP, this aspect is discussed in more detail below. Although MT1‐MMP degrades a variety of ECM components, it cannot degrade type IV collagen, a major component of the basement membrane (BM) [2, 5, 6].

### Soluble MMPs

**Fig. 2:**
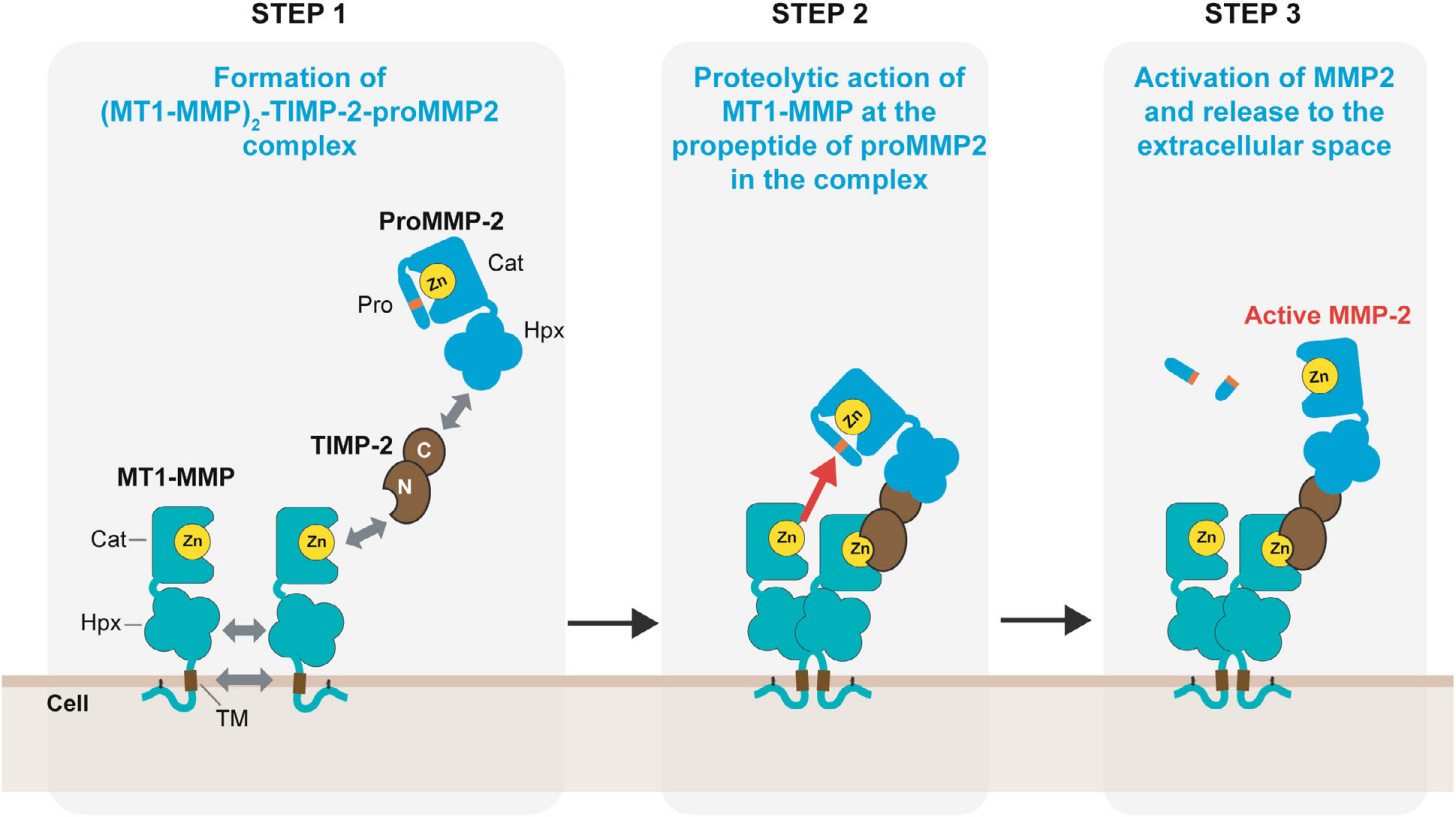
Current model of cell surface proMMP2 activation. Step 1: MT1‐MMP forms a homodimer through the interaction of the Hpx domain and the TM domain with each other. TIMP‐2, an endogenous MMP inhibitor, inhibits one of the MT1‐MMPs in the dimer through the N‐terminal inhibitory domain and binds the Hpx domain of proMMP2 at the C‐terminal domain, forming (MT1‐MMP)_2_‐TIMP‐2‐proMMP‐2 ternary complex. Step 2: MT1‐MMP that is not inhibited by TIMP‐2 cleaves the propetide of proMMP2 in the complex, inducing an intermediate active form of MMP2. Step 3: The Intermediate active form of MMP2 cleaves the rest of the propeptide each other and convert to the active form. Active MMP2 generated is then released from the cell surface.

MT1‐MMP activates other soluble proMMPs on the cell surface, namely proMMP2 and proMMP13, expanding the proteolytic repertoire within the cellular microenvironment [5]. In particular, proMMP2 activation is considered a crucial step during the initial invasion of epithelial cancer cells into the BM [15]. BM is a thin, sheet‐like structure composed of type IV collagen, laminin, nidogen, and perlecan, and it has been considered that the degradation of type IV collagen is a crucial step for cancer cells to invade the BM. The enzymes that degrade type IV collagen include MMP2, MMP3, MMP7, MMP9, MMP10, and MMP12 [6, 16, 17]. Among them, MMP2 was considered a crucial enzyme in cancer invasion, and the active form of MMP2 was detected in bladder carcinoma, which correlated with tumor grade and invasion [18]. Interestingly, the cells producing MMP2 were not cancer cells, but surrounding stromal cells [18], and thus, MT1‐MMP expressed by cancer cells activated proMMP2 produced by neighboring fibroblasts to invade BM [15]. A currently accepted model of proMMP2 activation is depicted in Fig. 2. In this model, MT1‐MMP forms a homodimer through the Hpx and TM domains, and one of the MT1‐MMP in the dimer is inhibited by its endogenous inhibitor, tissue inhibitor of metalloproteinases 2 (TIMP‐2). TIMP‐2 consists of an N‐terminal inhibitory domain and a C‐terminal domain that has a high affinity to the Hpx domain of proMMP2. Thus, (MT1‐MMP)_2_‐TIMP‐2 complex serves as a cell surface receptor for proMMP2. When proMMP2 binds to TIMP‐2 in the complex, the free MT1‐MMP, which is not bound to TIMP‐2, cleaves the propeptide of proMMP2, thereby inducing activation [5].

MT1‐MMP also activates proMMP13 on the cell surface [19]. It was reported that the Hpx domain of proMMP13 is essential for the activation, but it is not known how the Hpx domain is involved in the activation [5, 20]. This activation does not require TIMP‐2, and it is not clear if the dimerization of MT1‐MMP is required for the activation.

### Membrane proteins

It has been demonstrated that MT1‐MMP sheds the ectodomain of CD44, which promotes the migration of cancer cells [5, 21]. CD44 is a type I transmembrane hyaluronan (HA) receptor, and its N‐terminal globular domain contains an HA‐binding site [22]. In migrating cancer cells, CD44 and MT1‐MMP interact and localize at the migration front, where the shedding occurs [21, 23]. Thus, shedding enables the timely disengagement of CD44‐mediated by cell attachment at the leading edge, thereby enhancing cell migration [5].

MT1‐MMP was shown to shed syndecan‐1 and promote HT1080 cell migration [24]. Syndecans are type I transmembrane proteoglycans, and it has been shown that syndecan‐1 binds and activates αvβ3 and αvβ5 integrins [25]. The mechanism by which syndecan‐1 shedding promotes cell migration is not clearly understood [26]; however, it is possible that the shedding may influence integrin‐mediated cell adhesion.

Integrin alpha‐V is expressed as an immature single‐chain form and requires cleavage by proprotein convertases, such as furin, to convert into a two‐chain form consisting of a heavy chain and a light chain interconnected by a disulfide bond, thereby becoming a functionally active form. However, when MT1‐MMP is expressed, MT1‐MMP cleaves alternative sites and converts Integrin alpha‐V to be more efficient in adhesion and activating focal adhesion kinase, thereby increasing cell motility on Vitronectin [27, 28].

Endothelial cells express two receptors for vascular endothelial growth factor (VEGF), VEGFR1 and VEGFR2. They are closely related receptor tyrosine kinases (RTK), but VEGFR‐1 is a kinase‐impaired RTK and is considered a decoy receptor [29, 30]. MT1‐MMP was found to shed VEGFR1, but not VEGFR2; thus, MT1‐MMP promotes angiogenesis by inactivating a decoy receptor [31].

MT1‐MMP also cleaves the extracellular domain of the parathyroid hormone 1 receptor (PTH1R) in hypertrophic chondrocyte‐derived osteoblast lineage, thereby dampening PTH signaling [32]. HC‐lineage‐specific MT1‐MMP deficiency resulted in enhanced PTH signaling and higher bone production, suggesting that MT1‐MMP controls proper PTH/PTH1R signaling [32].

MT1‐MMP gene knockout in mice results in craniofacial abnormalities, attributed to a defect in FGFR2 (fibroblasts growth factor receptor 2) signaling [33]. It was found that MT1‐MMP forms a complex with FGFR2 and ADAM9 (a disintegrin and metalloprotease 9) in osteoblasts, proteolytically inactivating ADAM9 and thereby protecting FGFR2 from shedding [33]. MT1‐MMP is thus a critical negative modulator of ADAM9 to maintain FGFR2 signaling in calvarial osteogenesis [33].

MT1‐MMP gene knockout in mice was found to increase in hematopoietic progenitor cells and specifically impairs B‐lymphocyte development [34]. It was found that MT1‐MMP is expressed in bone marrow stromal cells and directly cleaves Dll1 to negatively regulate Notch signaling to specifically maintain normal B‐cell development in bone marrow [34].

In different epithelial cancer, both MT1‐MMP and EphA2 (Eph Receptor A2) are upregulated. It was shown that MT1‐MMP cleaved the ectodomain of EphA2 at the fibronectin type III domain 1 [35]. This cleavage was shown to result in intracellular EphA2 translocation as well as an increase in RhoA activity and cell junction disassembly, which suggests an overall increase in the repulsive effect between cells, triggering single‐cell breast cancer invasion [35]. Interestingly, in ovarian tumor tissue, a significant portion of EphA2 lost its N‐terminal portion in the cells that express MT1‐MMP [36]. Expression of EphA2 and MT1‐MMP was shown to promote ErbB signaling, anchorage‐independent growth, and cell migration [36]. When a noncleavable EphA2 mutant was overexpressed, tumorigenesis and metastasis of human cancer cells in a mouse xenograft model were effectively prevented, suggesting a crucial role of EphA2 cleavage by MT1‐MMP during cancer progression [36].

Taken together, MT1‐MMP is a powerful signaling modifier that influences cell fate and disease progression.

## MT1‐MMP and fibrillar collagen

### Fibrillar collagen maintains tissue architecture

There are seven fibrillar collagens in humans, including types I, II, III, V, XI, XXIV, and XXVII [37]. They have three chains of an uninterrupted Gly‐Xaa‐Yaa‐ repeat sequence of more than 1000 residues, intertwined, forming a tight triple helix. The monomer collagens then form a fibril structure, and their structures are further strengthened by crosslinking. Type I collagen is the most abundant collagen in vertebrates and consists of two *α*1(I) chains and one *α*2(I) chain. They are found in bone, skin, and most stromal tissues, providing tissues and organs with a shape and tensile strength. Its tissue levels are precisely controlled by fibroblast production and collagenase‐mediated proteolytic degradation. Over‐deposition of type I collagen is the common cause of fibrosis, while overdegradation would lead to tissue destruction. Therefore, collagen degradation is a key step in regulating collagen levels in tissues.

### MT1‐MMP maintains stromal collagen homeostasis

As discussed above, among the five fibrillar collagen‐degrading MMPs, namely MMP1, MMP2, MMP8, MMP13, and MT1‐MMP, MT1‐MMP is the only membrane‐bound collagenolytic MMP. The importance of MT1‐MMP as a collagenase is highlighted by the phenotype of *mmp14* null mice [38], displaying skeletal development defects, osteoclast‐mediated arthritis, and general fibrosis of stromal tissues. Stromal tissue fibrosis was not observed in any other collagenase MMP knockout mice, such as *mmp2, mmp8,* and *mmp13* null mice, suggesting that MT1‐MMP plays an essential role in collagen catabolism in stromal tissue [39, 40, 41]. In stromal tissue, type I collagen is constitutively produced by fibroblasts, and excess collagen is degraded by MT1‐MMP, maintaining homeostasis of stromal collagen levels. Thus, the absence of MT1‐MMP leads to the accumulation of excess collagen, resulting in fibrosis [38].

There is a human version of MT1‐MMP loss‐of‐function genetic disorder, Winchester syndrome [42]. Winchester syndrome is a rare inherited connective tissue disorder characterized by severe osteolysis (bone loss) in the hands and feet, osteoporosis, joint contractures, and coarse facial features [43]. One of the notable phenotypes of the disease is dermal fibrosis, characterized by abnormal thickening and scarring of the skin and connective tissues [43], which resembles the phenotype of MT1‐MMP null mice [38]. In Winchester syndrome, a homozygous single nucleotide mutation in the *MMP14* gene was identified, resulting in the substitution of an amino acid within the hydrophobic signal peptide (p.Thr17Arg) [42]. This mutation prevents MT1‐MMP from interacting with signal peptidase, leading to defects in signal peptide processing and severely decreased mature enzyme expression on the cell surface [42]. Therefore, Winchester syndrome is a human version of the MT1‐MMP null phenotype.

There is another example of a human disease associated with MT1‐MMP defect, Dupuytren's disease (DD) [44]. DD is a common fibroproliferative disease of the palmar fascia, affecting approximately 4% of the general population. DD begins with the initial appearance of nodules, followed by the formation of thickened contractile cords due to fibrosis, which eventually results in impaired hand function. It was found that a single nucleotide polymorphism (SNP) variant of MT1‐MMP (rs1042704, pD273N) was associated with DD, and the SNP variant of MT1‐MMP (MT1‐N273) was found to exhibit only 17% of cell surface collagen‐degrading activity compared to the wild‐type MT1‐MMP. About 45% of DD patients have the SNP allele. It has been thus concluded that the SNP (rs1042704) contributes to the fibrotic phenotype of DD [44].

Taken together, the phenotypes of null mice, Winchester syndrome, and DD highlight the crucial role of MT1‐MMP in maintaining homeostasis of stromal collagen matrix *in vivo*.

### MT1‐MMP is a promotor of collagen invasion and tissue destruction

Fibrillar collagen is the major component of the stromal tissue matrix, and when cells migrate, it becomes a major physical barrier that limits their movement. Under these conditions, cells create a migration path by degrading it using MMP collagenases. It has been shown that MT1‐MMP is the only collagenase that promotes cell invasion into the collagen matrix [45, 46, 47]. It was shown that expression of other collagenases did not promote collagen invasion, even though they are mutated to be activated intracellularly [46]. For MT1‐MMP to promote collagen invasion, MT1‐MMP must be anchored to the plasma membrane, as the expression of a transmembrane/cytoplasmic domain deletion mutant failed to promote the invasion [45, 46]. Interestingly, membrane‐anchored MMP13 even failed to degrade collagen on the cell surface, suggesting that cell surface collagen degradation activity is unique to MT1‐MMP, which is a homodimer formation through the Hpx domain [48]. Cell surface collagen degradation requires not only intrinsic collagenolytic activity, but also homodimerization on the cell surface [48]. MT1‐MMP‐mediated collagen invasion was also shown to be crucial for cancer growth within collagen matrix [46]. Thus, MT1‐MMP is thought to be a crucial player in cancer progression: invasion, metastasis, and growth.

In rheumatoid arthritis (RA), the inflamed synovial pannus invades cartilage and destroys joint tissue. Within pannus tissue, synovial fibroblasts are the primary cell type that invades cartilage. Major components of cartilage are type II collagen (45%) and aggrecan proteoglycans (45%), where type II collagen forms a cartilage architecture and proteoglycan fills the gaps between the collagen fibrils, drawing the water molecules within the cartilage. It was shown that cartilage invasion of synovial fibroblast is MT1‐MMP‐dependent [49], and selective inhibition of MT1‐MMP in a mouse model of arthritis inhibited cartilage erosion [50], suggesting that MT1‐MMP is indeed the cause of cartilage erosion.

## MT1‐MMP regulation

### Collagen‐mediated MT1‐MMP activation

MT1‐MMP is expressed in various cell types, including fibroblasts, osteoblasts, osteoclasts, chondrocytes, epithelial cells, endothelial cells, adipocytes, monocytes, macrophages, T cells, B cells, neuronal cells, and different cancer cells [4, 5]. However, little is known about the regulatory mechanisms of MT1‐MMP gene *in vivo*. One of the potential *in vivo* stimuli of MT1‐MMP gene expression and function is fibrillar collagen. It has been shown that fibroblasts, epithelial cells, endothelial cells, and some cancer cells upregulate MT1‐MMP expression and function when they are stimulated or cultured within a fibrillar collagen matrix [51, 52, 53, 54, 55, 56]. Since MT1‐MMP gene expression is poorly regulated by soluble factors such as cytokines and growth factors, and because fibrillar collagen is a major substrate of MT1‐MMP, this collagen‐mediated MT1‐MMP upregulation has been considered a major *in vivo* expression pathway. It was shown that collagen‐induced expression of MT1‐MMP in human fibroblasts is through the activation of a collagen receptor tyrosine kinase, discoidin domain receptor 2 (DDR2) [57]. In RA joints, MT1‐MMP is highly expressed in the synovial fibroblasts attached to the cartilage [49, 57]. Thus, it is likely that cartilage collagen is the stimulus for the MT1‐MMP expression in the RA joint. Interestingly, it was found that aggrecan‐removed cartilage stimulated the MT1‐MMP more effectively than intact cartilage [57]. In RA cartilage, aggrecan depletion precedes collagen degradation, and aggrecan removal may be a trigger of DDR2 activation. Thus, in RA joints, cartilage collagen is a stimulus as well as a substrate of MT1‐MMP in synovial fibroblasts. In stromal tissue, fibroblasts are the major producers of type I collagen, and a lack of MT1‐MMP was shown to result in stromal fibrosis [38, 42]. Thus, collagen produced by fibroblasts may stimulate MT1‐MMP gene expression and activity via DDR2 activation, which, in turn, degrades overproduced collagen, maintaining collagen homeostasis in the tissue. Fibrillar collagen also stimulates the MT1‐MMP gene and activity in some cancer cells, but it was shown that DDR2 is not involved in this activation [57], but rather integrin is involved in MT1‐MMP activation [58, 59]. However, the exact mechanism is still to be investigated.

### Cell surface localization of MT1‐MMP

**Fig. 3:**
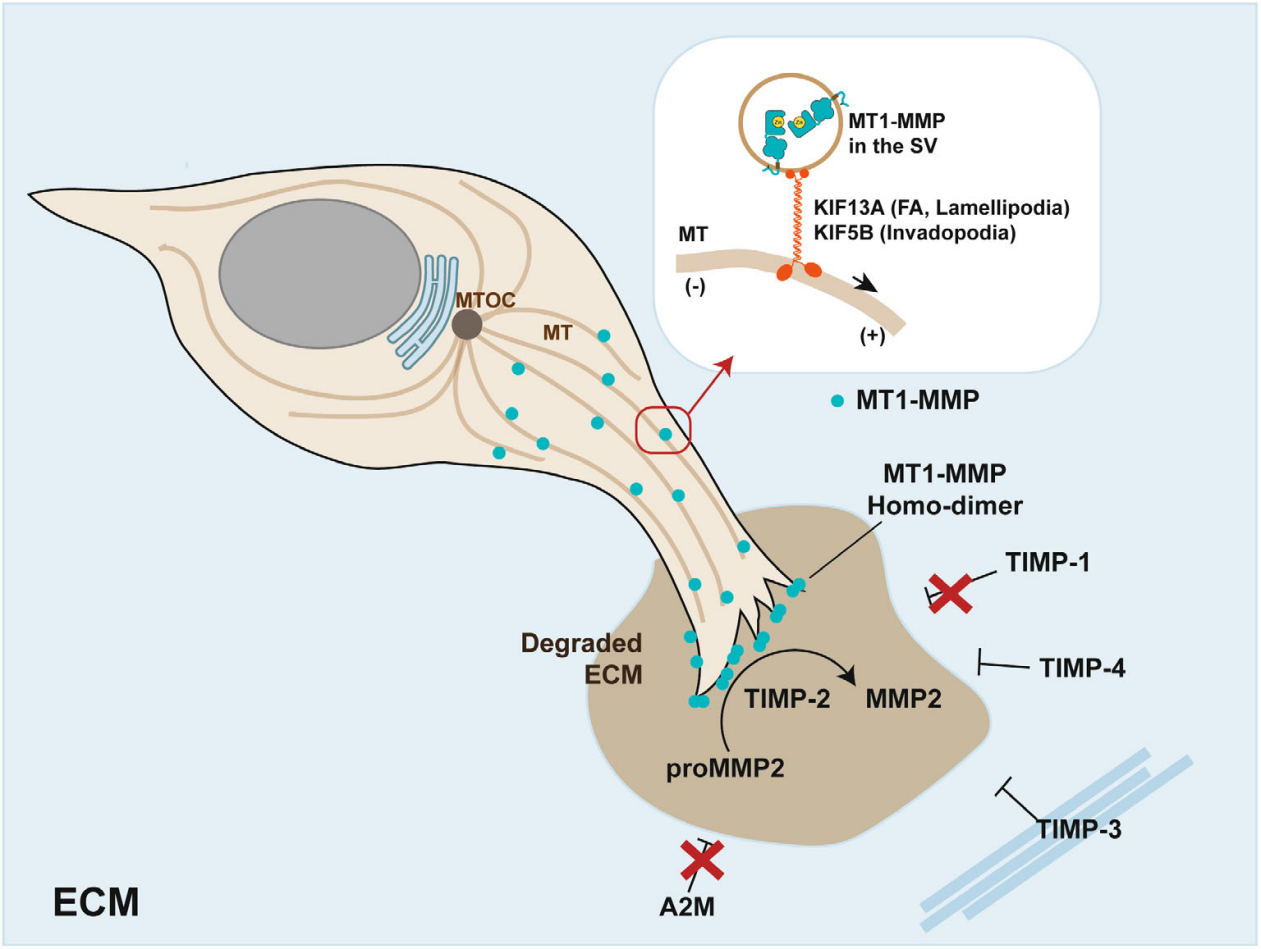
Regulation of MT1‐MMP during cell invasion and tissue destruction. MT1‐MMP synthesized in the cells is transported to the leading edge of invading cells along the microtubules within secretory vesicles by the action of N‐kinesin motor proteins, KIF13A (to FA and lamellipodia) or KIF5B (to invadopodia). This KIF‐mediated targeted vesicle transport is crucial for localizing MT1‐MMP at the leading edge, thereby promoting polarized ECM degradation. At the leading edge, MT1‐MMP forms a homodimer, thereby activating proMMP2 and degrading fibrillar collagens. In the tissue, the general proteinase inhibitor A2M and the endogenous MMP inhibitor TIMP1 are abundantly present, but they do not inhibit MT1‐MMP. TIMP‐2, another abundantly expressed MMP inhibitor, facilitates MT1‐MMP‐mediated activation of proMMP2 at the cell surface. TIMP‐3 and TIMP‐4 inhibit MT1‐MMP activity, but TIMP‐3 is present as ECM‐bound and tightly regulated by endocytosis. TIMP‐4 is also expressed in particular tissues. Thus, these TIMPs can regulate MT1‐MMP‐mediated cell invasion and tissue destruction only at their sites of expression. (−)/(+), minus‐/plus‐end of microtubules; A2M, alpha 2 macroglobulin; FA, focal adhesion; SV, secretory vesicle; KIF, kinesin superfamily proteins; MT, microtubules; MTOC, microtubule organization center; TIMP, tissue inhibitor of metalloproteinase.

To utilize MT1‐MMP, cells must localize it to specific plasma membrane sites, a process that is among the most essential post‐translational regulatory mechanisms of MT1‐MMP. When this mechanism is disturbed, cells lose invasive activity. MT1‐MMP has been shown to localize at the leading edge membrane structures, including lamellipodia, invadopodia, and focal adhesion (Fig. 3) [5]. Lamellipodia is the leading edge structure typically found in cells migrating on a 2D surface, and Rac1 small GTPase regulates the formation. Cells in a 3D matrix can still form lamellipodia, but their formation is influenced by the surrounding matrix [60]. In motile cancer cells, ruffling membranes form at the lamellipodial edge, a process that is markedly enhanced by Rac1 activation. MT1‐MMP was shown to localize highly to the ruffling membrane in cancer cells [61]. One of the mechanisms of MT1‐MMP localization at the lamellipodium is interaction with CD44, a hyaluronan receptor [23]. The cytoplasmic domain of CD44 binds to ERM proteins (Ezrin, Radixin, and Moesin) that interact with actin filaments at lamellipodia, allowing CD44 to localize at lamellipodia [62]. MT1‐MMP interacts with CD44 via the Hpx domain, thereby enabling MT1‐MMP localization and indirect association with fibrillar actin at lamellipodia [23]. It was also reported that MT1‐MMP may directly interact with radixin, one of the ERM proteins, through its cytoplasmic tail [63, 64]. However, it needs further confirmation.

MT1‐MMP has also been shown to localize at invadopodia [65, 66, 67]. Invadopodia were originally defined as membrane protrusions that extend toward the ECM and contain proteolytic enzymes that degrade the ECM in invasive cancer cells [68]. Subsequently, it was found that the molecular components of podosomes in myeloid cells are similar to those of invadopodia, and the two structures are now collectively referred to as invadosomes [67, 69]. MDA‐MB231 cells, a breast cancer cell line, were shown to exclusively localize MT1‐MMP at invadopodia and degrade collagen in an invadopodia‐dependent manner [70]. Invadopodia have been defined as membrane structures containing fibrillar actin, cortactin, Tks5, and MT1‐MMP that extend into the ECM under 2D culture conditions [66, 67]. However, its definition has been broadened not only to include the protrusions detected in 2D culture conditions but also flat membrane structures degrading ECM under 3D culture conditions as long as the F‐actin, cortactin, Tks5, and MT1‐MMP are present [70]. MT1‐MMP localization at invadopodia has been shown to result from the targeted transport of MT1‐MMP‐containing vesicles along microtubules, and KIF3A and KIF5 motor proteins have been implicated [71, 72]. However, other cancer cell lines, such as human fibrosarcoma HT1080 or human squamous cell carcinoma A431 cells, have been shown to degrade ECM by localizing MT1‐MMP to other membrane structures, such as focal adhesions [73, 74, 75].

Focal adhesion is the part of the plasma membrane that is formed by clusters of integrins attaching to the ECM [76]. Within the plasma membrane, focal adhesion is the closest to ECM, and it plays a crucial role in cell adhesion and transmitting microenvironmental signals to the cells through integrins [76]. It was found that inhibition of MT1‐MMP effectively stabilized focal adhesions, whereas overexpression of MT1‐MMP destabilized them, suggesting that MT1‐MMP is localized at the membrane structures and modulates the integrity of focal adhesions by degrading ECM [73, 74]. In HT1080 cells, disrupting MT1‐MMP localization to focal adhesions effectively inhibited collagen invasion [74], suggesting that focal adhesions may function as a leading edge of invading cells. Localization of MT1‐MMP at focal adhesions is also mediated by the targeted transport of MT1‐MMP‐containing vesicles along microtubules, and KIF3A and KIF13A have been shown to coordinate vesicle transfer to focal adhesions [75]. Since inhibition of KIF13A‐mediated vesicle transport of MT1‐MMP also inhibited lamellipodia localization of MT1‐MMP [75], it is possible that the same mechanism may be applied to lamellipodia localization (Fig. 3).

### Endocytosis

After its appearance on the cell surface, MT1‐MMP remains there for a period of time before being endocytosed. MT1‐MMP on the cell surface is thus constantly replaced by the newly synthesized enzyme. The half‐life at the cell surface depends on the mode of endocytosis. MT1‐MMP has been shown to be internalized via a clathrin‐mediated pathway [77, 78], which has the fastest turnover, and its half‐life on the cell surface can be less than 30 min [77, 79]. The ^575^LLY motif in the cytoplasmic tail was shown to interact with μ2 subunit of adapter protein‐2 of clathrin [77]. Endocytosis can be considered a negative regulatory mechanism; however, interfering with clathrin‐mediated endocytosis severely inhibited the cell migration‐promoting effect of MT1‐MMP [77]. It has also been shown that palmitoylation of the Cys^574^ is essential for clathrin‐mediated endocytosis [79], suggesting that the position of ^575^LLY relative to the plasma membrane may be important for the incorporation of MT1‐MMP to the clathrin‐coated pits. MT1‐MMP was also shown to be internalized via a caveolae‐mediated manner [78, 79]. This pathway has a much slower turnover, with a cell surface half‐life of approximately 60 min [79]. Caveolae‐mediated endocytosis can be detected upon inhibition of clathrin‐mediated endocytosis, accompanied by loss of the cell migration‐promoting effect. Thus, caveolae‐mediated endocytosis may not support MT1‐MMP function in some cell types.

### Inhibition by endogenous inhibitors

As a member of MMPs, MT1‐MMP can be inhibited by tissue inhibitors of metalloproteinases (TIMPs). There are four TIMPs, TIMP‐1, TIMP‐2, TIMP‐3, and TIMP‐4 [14]. Among them, TIMP‐1 and TIMP‐2 are widely expressed in tissues and plasma [80, 81], whereas TIMP‐4 is restricted to the brain, cardiovascular tissues, and adipose tissue [82, 83]. TIMP‐3 is also expressed broadly but has a high affinity for sulphated ECM, including cell surface proteoglycans, and its availability is tightly controlled by the scavenger receptor LRP‐1 (low‐density lipoprotein receptor‐related protein 1) [84, 85]. Among these TIMPs, TIMP‐1 cannot inhibit MT1‐MMP at physiological concentrations [86]. Thus, MT1‐MMP can function under the TIMP‐1‐enriched tissue environment. TIMP‐2 can inhibit MT1‐MMP; however, TIMP‐2 is essential for one of MT1‐MMP's functions, namely, proMMP‐2 activation, as described above. TIMP‐3 is considered the major MT1‐MMP inhibitor in tissues, although its availability is tightly regulated by endocytosis [84, 85]. In TIMP‐3‐deficient conditions, MT1‐MMP‐mediated activation by proMMP‐2 was enhanced, supporting the notion [87]. TIMP‐4 is another potential MT1‐MMP inhibitor, as loss of TIMP‐4 leads to increased atherosclerotic plaque deposition in the abdominal aorta [88], which may be attributed to a defect in MT1‐MMP inhibition.

Another general proteinase inhibitor that inhibits multiple MMPs and other classes of proteinases is alpha‐2‐macroglobulin (A2M) [89]. It is 725 kDa in size and consists of four identical subunits. A2M possesses a ‘bait region’ where different proteinases can cleave, and cleavage of the bait region induces massive structural alteration, entrapping the proteinase inside of the four subunits, which keeps the proteinase away from macro substrates [89]. It is a major inhibitor for all classes of proteinases present in the plasma, and there is no doubt that A2M can inhibit soluble recombinant MT1‐MMP in solution. However, it is unlikely that A2M inhibits membrane‐bound MT1‐MMP, as it would not be able to entrap type I transmembrane molecules. Thus, cells expressing MT1‐MMP likely utilize MT1‐MMP in the presence of A2M, which inhibits other soluble proteinases (Fig. 3). In fact, MT1‐MMP can enhance cellular invasion and matrix degradation in serum and *in vivo*. GPI‐anchored glycoprotein RECK has been shown to inhibit MT1‐MMP [90], but its role as a regulator of MT1‐MMP remains unclear [91].

## Conclusion and future prospects

As discussed, MT1‐MMP modifies the immediate cellular microenvironment by cleaving pericellular ECM. Additionally, it modulates the cellular response to microenvironmental cues by cleaving cell surface receptors, suggesting that it is an effective modulator of microenvironmental signals. MT1‐MMP is a critical enzyme in development and collagen homeostasis as suggested by the phenotypes of MT1‐MMP null mice and MT1‐MMP‐related human diseases (Winchester syndrome and DD). At the same time, MT1‐MMP also promotes tissue‐destructive diseases such as cancer and arthritis by promoting cellular invasion. Tissue destruction can occur via two mechanisms: ECM degradation by soluble proteinases and cell invasion mediated by MT1‐MMP. A former example is aggrecan degradation in osteoarthritis cartilage, which is caused by soluble ADAMTS (a disintegrin and metalloproteinase with thrombospondin motifs) proteinases, such as ADAMTS4 and 5, produced by chondrocytes [92, 93]. On the other hand, in RA and cancer tissue destruction is due to the cellular invasion, attributed to MT1‐MMP activity. Thus, MT1‐MMP has been considered as a potential therapeutic target for these diseases. In the 1990s, clinical trials of small‐molecule metalloproteinase inhibitors failed, attributed to their broad inhibitory activity of the inhibitors. There are 62 metalloproteinases in humans, and these inhibitors broadly inhibit many of these enzymes, causing musculoskeletal syndromes. In 2009, a highly selective MT1‐MMP antibody inhibitor, DX‐2400, was engineered and demonstrated to effectively inhibit cancer invasion, angiogenesis, and metastasis in preclinical experiments [94]. DX‐2400 also effectively inhibited the progression of inflammatory arthritis in a mouse model [50]. It is a highly selective, if not specific, inhibitor and did not exhibit the side effects observed with small‐molecule metalloproteinase inhibitors [94]. Thus, it was considered very promising. However, due to the previous failure of metalloproteinase inhibitor clinical trials, pharma companies refused to further develop it. Given the significance of MT1‐MMP in collagen homeostasis, it may not be advisable to inhibit MT1‐MMP systemically in the long term. Ideally, MT1‐MMP should be explicitly targeted in disease‐causing cells. Recently, it has become clearer that its cellular regulation is critical for MT1‐MMP function, and interfering with its mechanism can result in functional inhibition of MT1‐MMP without directly inhibiting its activity [2]. Thus, identifying druggable targets to modulate MT1‐MMP regulation and suppress cell invasion may be a promising approach for developing future anti‐cell‐invasion therapies. In addition, the Human Cell Atlas initiative is now a major global challenge [95], and it may be possible to further profile disease‐causing cell types in more detail in the future. In combination with this information, it may be possible to develop cell‐specific anti‐invasion therapies in the future. It would be of interest to further understand the cellular regulatory mechanisms of MT1‐MMP and to develop the Human Disease Cell Atlas in the future.

## Author contributions

YI wrote the manuscript. YI and MI edited the manuscript.

## Conflict of interest

The authors declare no conflict of interest.
